# Identification of key molecular markers of acute coronary syndrome using peripheral blood transcriptome sequencing analysis and mRNA-lncRNA co-expression network construction

**DOI:** 10.1080/21655979.2021.2003932

**Published:** 2021-12-29

**Authors:** Ming Shen, Rui Gong, Haibin Li, Zhihui Yang, Yunpeng Wang, Dandan Li

**Affiliations:** a Department of Cardiology, The First Hospital of Hebei Medical University, Shijiazhuang, China; b Department of Internal medicine-Endocrinology, Children’s Hospital of Hebei, Shijiazhuang, China; c Department of General Medicine, The Third Hospital of Hebei Medical University, Shijiazhuang, China

**Keywords:** Acute coronary syndrome, mRNA, long non-coding RNA, differential expression, diagnostic

## Abstract

Acute coronary syndrome (ACS) is a term used to describe major cardiovascular diseases, and treatment of in-stent restenosis in patients with ACS remains a major clinical challenge. Further investigation into molecular markers of ACS may aid early diagnosis, and the treatment of ACS and post-treatment recurrence. In the present study, total RNA was extracted from the peripheral blood samples of 3 patients with ACS, 3 patients with percutaneous coronary intervention (PCI)_non-restenosis, 3 patients with PCI_restenosis and 3 healthy controls. Subsequently, RNA library construction and high-throughput sequencing were performed. DESeq2 package in R was used to screen genes that were differentially expressed between the different samples. Moreover, the intersection of the differentially expressed mRNAs (DEmRNAs) and differentially expressed long noncoding RNAs (DElncRNAs) obtained. GeneCodis4.0 was used to perform function enrichment for DEmRNAs, and lncRNA-mRNA co-expression network was constructed. The GSE60993 dataset was utilized for diagnostic analysis, and the aforementioned investigations were verified using in vitro studies. Results of the present study revealed a large number of DEmRNAs and DElncRNAs in the different groups. We selected genes in the top 10 of differential expression and also involved in the co-expression of lncRNA-mRNA for diagnostic analysis in the GSE60993 dataset. The area under curve (AUC) of *PDZK1IP1* (0.747), *PROK2* (0.769) and *LAMP3* (0.725) were all >0.7. These results indicated that the identified mRNAs and lncRNAs may act as potential clinical biomarkers, and more specifically, *PDZK1IP1, PROK2* and *LAMP3* may act as potential biomarkers for the diagnosis of ACS.

## Introduction

Acute coronary syndrome (ACS) describes a range of major cardiovascular diseases, which include acute myocardial infarction and unstable angina [1,2]. The morbidity and mortality rates associated with ACS remain high [3]. Percutaneous coronary intervention (PCI) combined with stent implantation can improve the prognosis of patients with ACS [4]. However, the treatment of in-stent restenosis in patients with ACS remains a major clinical challenge [5]. Thus, further investigation into the expression and interaction of genes associated with ACS and post-treatment in-stent restenosis is of great significance for understanding the molecular mechanisms underlying ACS. Further investigations may provide a novel theoretical basis for the diagnosis and management of ACS.

Although the specific pathological mechanisms underlying ACS remain to be fully elucidated, multiple genes have been reported to be involved in its pathogenesis. mRNA mediates the translation of genetic information from genes into proteins, and plays an important role in disease progression and treatment [6]. Previous studies have demonstrated that abnormal mRNA expression plays an important regulatory role in the progression of ACS. ACS can be caused by inflammatory factors, and *macrophage migration inhibitory factor* (*MIF*) is an important regulator of inflammation. Compared with control group, the level of *MIF* in the serum of patients with ACS was significantly increased [7,8]. Moreover, *toll like receptor 2* (*TLR2*) is a member of the TLR superfamily, which coordinates platelet function when activated. Previous studies have demonstrated that the mRNA expression levels of *TLR2* in the platelets of patients with ACS were up-regulated, which may act as a potential biomarker [9]. In vitro, *NADH: ubiquinone oxidoreductase subunit C2* (*NDUFC2*) silencing affected vascular cell viability and angiogenesis. A significant decrease in *NDUFC2* expression was detected in ACS, which indicated that *NDUFC2* may play an important role in the development of ACS [10]. These studies highlight that differential expression of mRNA plays an important regulatory role in ACS.

Long noncoding RNAs (lncRNAs) are a class of non-coding RNAs ranging in length from ~200 nucleotides to 100 kilobases (kb) and are key components of gene regulatory networks [11,12]. Previous studies have shown that the dysregulation of lncRNA expression can lead to the development of cardiovascular diseases [13]. lncRNA *HLA complex group 11* (*HCG11*) regulates vascular endothelial cell proliferation and angiogenesis through the microRNA (miR/miRNA)-26b-5p/QKI-5 signaling pathway [14]. Moreover, lncRNA *antisense non-coding RNA INK4 locus* (*ANRIL*) has become an important risk factor for coronary disease due to its involvement in the regulation of histone methylation [15,16]. Significant changes in the expression of lncRNAs were observed during the development of ACS [2]. Moreover, knockdown of *ATPase plasma membrane Ca^2+^ transporting 1 antisense RNA 1* (*ATP2B1-AS1*) in mice may inhibit the nuclear factor-κB (NF-κB) pathway through the up-regulation of *nuclear factor-kappa-B inhibitor alpha* (*NFKBIA*) expression, thus playing an important role in the regulation of myocardial infarction [17]. miRNAs also play an important regulatory role in the progression of ACS [18]. miRNAs are a class of small non-coding RNAs that are involved in the regulation of gene expression [19]. Serum *miRNA-499, miRNA-210* and *miRNA-941* have potential diagnostic value in early diagnosis of ACS [20,21]. In addition, lncRNA *TCONS_00024652* regulates vascular endothelial cell proliferation and angiogenesis through *miRNA-21* [13]. Previous studies have revealed that lncRNAs play an important role in ACS disease progression by regulating mRNAs and miRNAs. However, the expression pattern of lncRNAs and their relative role in ACS require further investigation. Thus, analysis of gene transcriptome data of ACS is of great importance for further diagnosis and the development of novel treatment options.

Transcriptome sequencing (RNAseq) is a well-established method for analyzing the entire transcriptome, and is commonly used to evaluate the differential expression of genes in case-control studies [22]. However, research into the transcriptome of ACS and in-stent restenosis remains limited. Bioinformatics analyses such as functional enrichment analysis, Venn diagrams and correlation network construction are often used to identify key genes in diseases [23–27]. Thus, in order to determine key potential molecular markers for the early diagnosis and treatment of ACS and post-treatment in-stent restenosis, RNA-seq analysis was performed in the present study. The DESeq2 package was subsequently used for the screening of differentially expressed mRNAs (DEmRNAs) and differentially expressed lncRNAs (DElncRNAs). In order to further understand the biological functions of DEmRNAs, functional enrichment analysis was also performed in the present study. In order to identify genes associated with the development of ACS, lncRNA-mRNA co-expression network was developed. Subsequently, we selected the genes in the top 10 of differential expression and also involved in the co-expression of lncRNA-mRNA for diagnostic analysis to further evaluate the reliability of our results. Collectively, the results of the present study may provide novel insights into ACS and post-treatment in-stent restenosis, and may contribute to the development of novel diagnostic and therapeutic targets.

## Materials and methods

### Patients

The study population included 3 patients with ACS, 3 patients with PCI_non-restenosis (PCI_NR), 3 patients with PCI_restenosis (PCI_Re) and 3 normal healthy controls (NC). Detailed clinical information is displayed in Table 1. All patients aged between 56 and 79 years. Patients who met the definition of acute myocardial infarction and unstable angina were included in the ACS group [5]. Detailed exclusion criteria for patients with ACS were as follows: (1) patients who had been administered fish oil or drugs containing n-3 polyunsaturated fatty acids (n-3 PUFA) on admission; (2) patients who had been administered fish oil or received n-3 PUFA treatment following the onset of ACS; (3) patients who exhibited symptoms of active malignant diseases; (4) patients with liver dysfunction (aspartate aminotransferase>100 IU/L, alanine aminotransferase>100 IU/L) and severe renal insufficiency accompanied by hemodialysis. Patients enrolled in the present study initially received interventional treatment, and patients were included in the PCI_Re group following detection of severe in-stent restenosis using coronary angiography [28]. Patients who exhibited signs of restenosis that was not caused by interventional therapy were excluded from the present study. Moreover, patients in which restenosis was not detected following interventional treatment were included in the PCI_NR group. The individuals in the NC group were gender and age matched with the other groups, and they were healthy without any disease.

**Table 1.** Detailed information of sequencing samples

Group	Sample number	Gender	Age	Hypertension history	Diabetes history	Smoking history	Drinking history	Pressure conditions	Movement	Atherosclerosis history	Chest pain, chest tightness, arrhythmia, heart failure and other symptoms	Low Density Lipoprotein
Acute coronary syndrome (ACS) group	H1	Male	56	Yes	No	Yes	Yes	Big	Few	Yes	Yes	5.1 mmol/L
	H2	Female	70	No	Yes	No	No	Small	Normal	Yes	Yes	4.4 mmol/L
	H3	Male	67	Yes	Yes	Yes	Yes	Big	Few	Yes	Yes	3.8 mmol/L
Percutaneous coronary intervention_non-restenosis (PCI_NR) group	Z1	Female	60	Yes	Yes	No	No	Big	Few	Yes	No	4.6 mmol/L
	Z2	Male	75	Yes	No	Yes	No	Big	Few	No	Yes	5.7 mmol/L
	Z3	Male	59	No	Yes	Yes	Yes	Big	Few	No	Yes	4.1 mmol/L
PCI_restenosis (PCI_Re) group	X1	Female	63	No	No	No	No	Big	Normal	No	Yes	4.2 mmol/L
	X2	Female	71	No	Yes	No	No	Big	Few	Yes	No	5.4 mmol/L
	X3	Male	59	No	No	Yes	Yes	Small	Exercise	Yes	Yes	4.5 mmol/L
Normal controls (NC) group	C1	Female	61	No	No	No	No	Small	Normal	No	No	3.1 mmol/L
	C2	Male	78	No	No	Yes	No	Small	Normal	No	No	2.9 mmol/L
	C3	Female	79	No	No	No	No	Small	Normal	No	No	2.8 mmol/L

Group	High density lipoprotein	Total cholesterol	Triglyceride	Blood platelet count	Cardiactroponin (cTn)	Creatine kinase isoenzyme (CK-MB)	Electrocardiograph	Percutaneous coronary intervention (PCI) treatment	Time for stenosis after PCI	Coronary angiography results (percentage of restenosis in the original lesion)
Acute coronary syndrome (ACS) group	0.9 mmol/L	7.5 mmol/L	3.3 mmol/L	232	5.1 ug/L	189 U/L	ST-T changed			
	1.1 mmol/L	6.9 mmol/L	4.4 mmol/L	274	0.2 ug/L	24 U/L	ST-T changed			
	1.1 mmol/L	6.1 mmol/L	2.5 mmol/L	212	3.3 ug/L	131 U/L	ST-T changed			
Percutaneous coronary intervention_non-restenosis (PCI_NR) group	1.2 mmol/L	6.8 mmol/L	3.6 mmol/L	178	0.04 ug/L	12 U/L	ST-T changed	1 diagonal bracket and 1 right crown bracket		
	1.6 mmol/L	7.1 mmol/L	2.7 mmol/L	236	0.2 ug/L	13 U/L	ST-T changed	2 right crown stents		
	1.5 mmol/L	7.2 mmol/L	2.9 mmol/L	208	0.1 ug/L	18 U/L	ST-T changed	2 roundabout support bracket		
PCI_restenosis (PCI_Re) group	1.8 mmol/L	7.2 mmol/L	4.2 mmol/L	241	0.2 ug/L	15 U/L	ST-T changed	1 left main stent and 1 right proximal crown stent	12 months	100%
	1.0 mmol/L	8.2 mmol/L	3.4 mmol/L	203	0.2 ug/L	10 U/L	ST-T changed	2 anterior descending branches and 1 right crown	6 months	80%
	1.2 mmol/L	7.2 mmol/L	1.9 mmol/L	218	3.2 ug/L	61 U/L	ST-T changed	2 roundabout support bracket	1 month	99%
Normal controls (NC) group	1.6 mmol/L	5.3 mmol/L	2.3 mmol/L	189	0.03 ug/L	12 U/L	Normal			
	1.4 mmol/L	5.4 mmol/L	2.2 mmol/L	230	0.01 ug/L	8 U/L	Normal			
	1.8 mmol/L	5.6 mmol/L	1.9 mmol/L	285	0.01 ug/L	11 U/L	Normal			

### RNA library construction, sequencing, and rawdata processing

Total RNA was extracted from the peripheral blood samples of the participants. Illumina TruseqTM RNA Sample Prep kit was used to construct the chain specific library. Agilent 2100 BioAnalyzer and ABI StepOnePlus Real-Time PCR System were used to detect the quality of the library. The BGIseq platform was sequenced using the PE100 strategy. Fastp was used for quality control of the original sequencing data. The high quality sequence obtained following quality control was aligned to the human reference genome (GRCh38) in the Ensemble database [29] using the HISAT2 program (https://ccb.jhu.edu/software/hisat2/index.shtml) [30]. Expression of mRNAs and lncRNAs were normalized and outputted using stringtie (http://ccb.jhu.edu/software/stringtie/) [31].

### Differential analysis of mRNAs and lncRNAs

The DESeq2 package in R was used to screen genes with significant differences between samples (http://bioconductor.org/packages/DESeq2/) [32]. Firstly, standardize the original read count (mainly to correct the sequencing depth). Then, calculate the probability of hypothesis test (P-value) through the statistical model. Thirdly, multiple hypothesis testing correction (Benjiamini and Hochberg method) was performed to obtain the corrected p value (false discovery rate, FDR). Padj<0.05 and |log2 foldchange| (|log2FC|)>1 were used to the differential expression screening criteria of mRNAs and lncRNAs.

### Functional analysis of genes

Biological function enrichment analysis of DEmRNAs was performed. The Gene Ontology (GO) and Kyoto Encyclopedia of Genes and Genomes (KEGG) functional enrichment analyses were performed using the GeneCodis4.0 database (https://genecodis.genyo.es/) [33]. Pval_adj<0.05 was the screening standard.

### Analysis of lncRNA-mRNA co-expression relationship

The correlation between lncRNAs and mRNAs was analyzed using Pearson’s correlation coefficient. P value (P)<0.01 and |correlation coefficient| (|r|)≥0.8 for screening criteria. Then, GO and KEGG functional enrichment analysis were performed on the screened lncRNAs and mRNAs.

### Diagnostic analysis

The GSE60993 dataset containing 26 cases of ACS and 7 normal healthy controls was obtained from the Gene Expression Omnibus (GEO) database (http://www.ncbi.nlm.nih.gov/geo) [34], and the RNA sequencing type was mRNA. The aforementioned dataset was used for the diagnostic analysis of identified DEmRNAs. The receiver operating characteristic (ROC) analysis was also performed by using pROC package in R language. The sensitivity and specificity at the cutoffs were determined according to a previous study [35].

### RT-qPCR analysis

14 blood samples from 5 patients with ACS, 5 PCI_Re patients and 4 NC were obtained for in vitro validation. Total RNA was extracted from the blood samples using the RNAliquid Ultra-speed Whole Blood (liquid sample) kit (RN2602, Beijing Huitian Oriental Technology Co., Ltd.), according to the manufacturer’s protocols. The cDNA was synthesized using the FastKing cDNA first strand synthesis kit (KR116, TIANGEN), and then stored at −20°C or lower temperature. Real time-polymerase chain reaction (RT-PCR) was performed using SuperReal PreMix Plus (SYBR Green) kit (FP205, TIANGEN). *GAPDH* and *ACTB* were the internal reference of mRNA. The relative quantitative analysis of the data was performed by 2^−ΔΔCt^ method [36]. All experimental procedures were approved by The Third Hospital of Hebei Medical University (K2019-012-1).

### Statistical analysis

Results are presented as the mean ± standard deviation (SD). One-way ANOVA was used to statistically analyze the in vitro experiments. All experiments were independently repeated at least three times. P < 0.05 was considered to indicate a statistically significant difference.

## Results

ACS is one of the most serious cardiovascular diseases. The treatment of in-stent restenosis in ACS patients remains a major clinical challenge. In order to find potential key molecular markers to help early diagnosis and treatment of ACS and post-treatment recurrence, we performed transcriptome sequencing analysis. The study population included 3 patients with ACS, 3 PCI_NR patients, 3 PCI_Re patients and 3 NC. RNA was extracted from blood samples for library construction and high-throughput sequencing. DESeq2 package in R was used to screen genes with differentially expressed between different samples. Subsequently, functional enrichment analysis, lncRNA-mRNA co-expression network construction, diagnostic analysis and in vitro validation were performed. All results indicate that identified mRNAs and lncRNAs may be used as potential clinical biomarkers. In addition, we speculated that *PDZK1IP1, PROK2* and *LAMP3* may be used as the diagnosis of ACS.

### DEmRNAs analysis in PCI_NR/PCI_Re group

**Figure 1.**
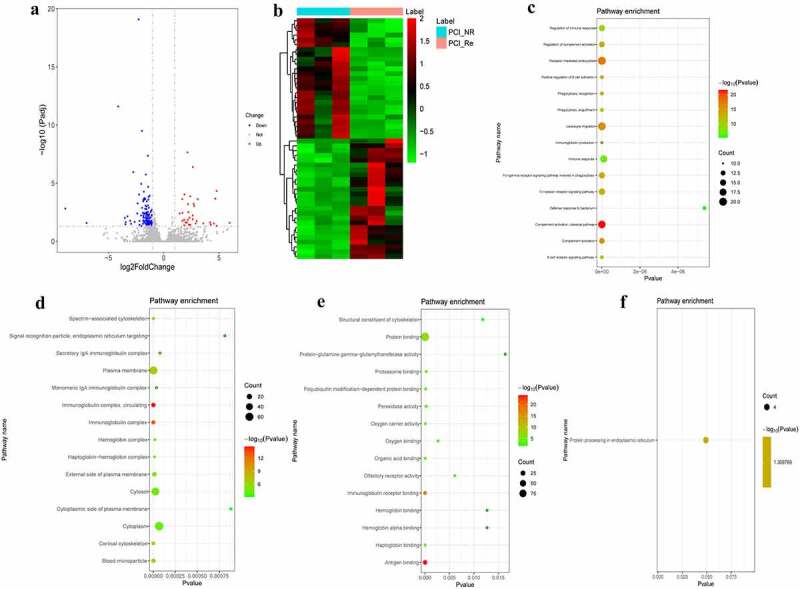
DEmRNAs analysis of the PCI_NR/PCI_Re group.

In order to reveal genes associated with in-stent restenosis (recurrence) in patients with ACS, differentially expressed genes were analyzed in the PCI_NR/PCI_Re group. According to the aforementioned screening criteria, there were 170 DEmRNAs (33 up-regulated and 137 down-regulated) in the PCI_NR/PCI_Re group. The volcano map of DEmRNAs is displayed in Figure 1a and a heat map of DEmRNAs is displayed in Figure 1b. To understand the biological functions of DEmRNAs, GO and KEGG functional analyses were performed (Pval_adj<0.05). The biological process (BP), cell composition (CC) and molecular function (MF) enrichment results of the top 15 are displayed in Figure 1c-e. Results of the KEGG enrichment analysis demonstrated that protein processing in the endoplasmic reticulum was the only significantly enriched signaling pathway (figure 1f).

a: Volcano map of DEmRNAs in the PCI_NR/PCI_Re group; b: Heatmap of DEmRNAs in the PCI_NR/PCI_Re group; c: Top 15 significantly enriched biological process (BP); d: Top 15 significantly enriched cell composition (CC); e: Top 15 significantly enriched molecular function (MF); f: Kyoto Encyclopedia of Genes and Genomes (KEGG) pathways. Heatmap shows result of a two-way hierarchical clustering of DEmRNAs (row) and samples (column). DEmRNAs clustering tree is displayed on the left. Red indicates above the reference channel. Green indicates below the reference channel.

### DEmRNAs analysis in NC/PCI_Re group

**Figure 2.**
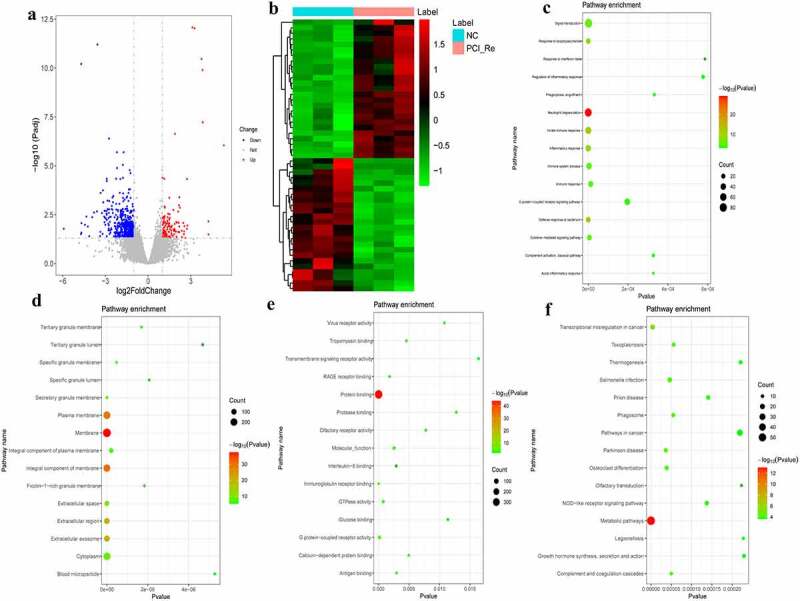
DEmRNAs analysis of the NC/PCI_Re group.

According to the aforementioned screening criteria, there were 635 DEmRNAs (124 up-regulated and 511 down-regulated) in the NC/PCI_Re group. The volcano map of DEmRNAs is displayed in Figure 2a. The heat map of DEmRNAs is displayed in Figure 2b. In order to understand the biological functions of DEmRNAs, GO and KEGG functional analyses were performed (Pval_adj<0.05). The BP, CC and MF enrichment results of the top 15 are displayed in Figure 2c-e. Results of the KEGG enrichment analysis demonstrated that metabolic pathways and transcriptional misregulation in cancer were significantly enriched signaling pathways (figure 2f).

a: Volcano map of DEmRNAs in the NC/PCI_Re group; b: Heatmap of DEmRNAs in the NC/PCI_Re group; c: Top 15 significantly enriched biological process (BP); d: Top 15 significantly enriched cell composition (CC); e: Top 15 significantly enriched molecular function (MF); f: Top 15 significantly enriched Kyoto Encyclopedia of Genes and Genomes (KEGG) pathways. Heatmap shows result of a two-way hierarchical clustering of DEmRNAs (row) and samples (column). DEmRNAs clustering tree is displayed on the left. Red indicates above the reference channel. Green indicates below the reference channel.

### DEmRNAs analysis in NC/ACS group

**Figure 3.**
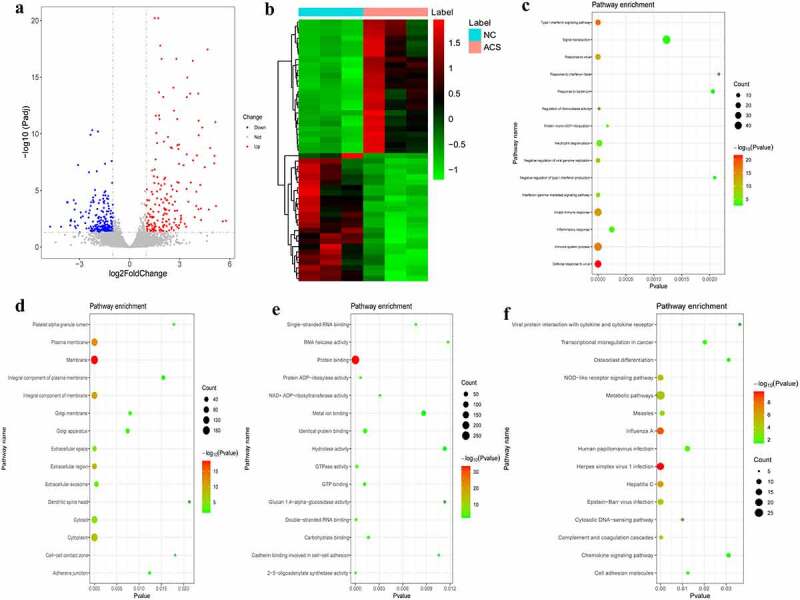
DEmRNAs analysis of the NC/ACS group.

According to the aforementioned screening criteria, there were 408 DEmRNAs (183 up-regulated and 225 down-regulated) in the NC/ACS group. The volcano map of DEmRNAs is displayed in Figure 3a. The heat map of DEmRNAs is displayed in Figure 3b. In order to understand the biological functions of DEmRNAs, GO and KEGG functional analyses were performed (Pval_adj<0.05). The BP, CC and MF enrichment results of the top 15 are displayed in Figure 3c-e. Results of the KEGG enrichment analysis demonstrated that herpes simplex virus 1 infection and influenza A were significantly enriched signaling pathways (figure 3f).

a: Volcano map of DEmRNAs in the NC/ACS group; b: Heatmap of DEmRNAs in the NC/ACS group; c: Top 15 significantly enriched biological process (BP); d: Top 15 significantly enriched cell composition (CC); e: Top 15 significantly enriched molecular function (MF); f: Top 15 significantly enriched Kyoto Encyclopedia of Genes and Genomes (KEGG) pathways. Heatmap shows result of a two-way hierarchical clustering of DEmRNAs (row) and samples (column). DEmRNAs clustering tree is displayed on the left. Red indicates above the reference channel. Green indicates below the reference channel.

### DEmRNAs analysis in ACS/PCI_NR group

**Figure 4.**
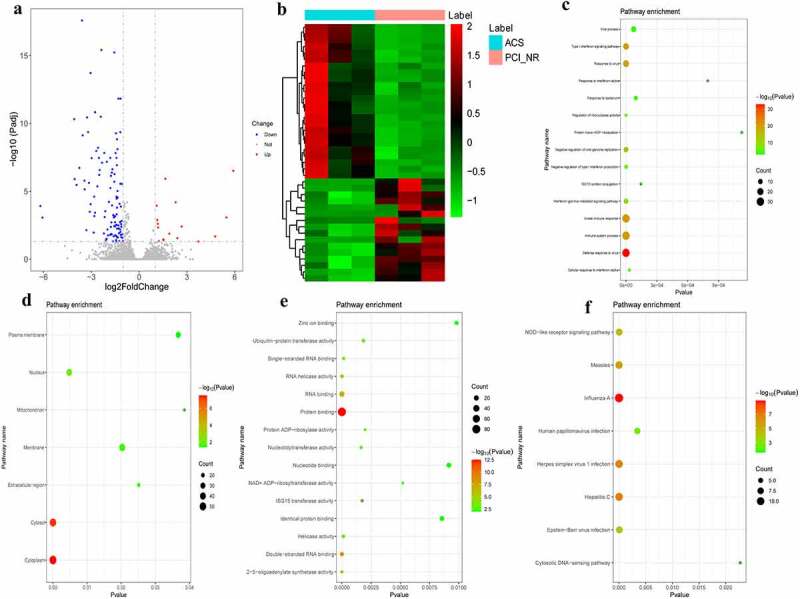
DEmRNAs analysis in ACS/PCI_NR group.

According to the aforementioned screening criteria, there were 127 DEmRNAs (16 up-regulated and 111 down-regulated) in the ACS/PCI group. The volcano map of DEmRNAs is displayed in Figure 4a. The heat map of DEmRNAs is displayed in Figure 4b. In order to understand the biological functions of DEmRNAs, GO and KEGG functional analyses were performed (Pval_adj<0.05). The BP, CC and MF enrichment results of the top 15 are displayed in Figure 4c-e. Results of the KEGG enrichment analysis demonstrated that influenza A and hepatitis C were significantly enriched signaling pathways (figure 4f).

a: Volcano map of DEmRNAs in the ACS/PCI_NR group; b: Heatmap of DEmRNAs in the ACS/PCI_NR group; c: Top 15 significantly enriched biological process (BP); d: Cell composition (CC); e: Top 15 significantly enriched molecular function (MF); f: Kyoto Encyclopedia of Genes and Genomes (KEGG) pathways. Heatmap shows result of a two-way hierarchical clustering of DEmRNAs (row) and samples (column). DEmRNAs clustering tree is displayed on the left. Red indicates above the reference channel. Green indicates below the reference.

### Analysis of DEmRNAs of the intersection of the PCI_NR/PCI_Re and NC/PCI_Re groups

**Figure 5.**
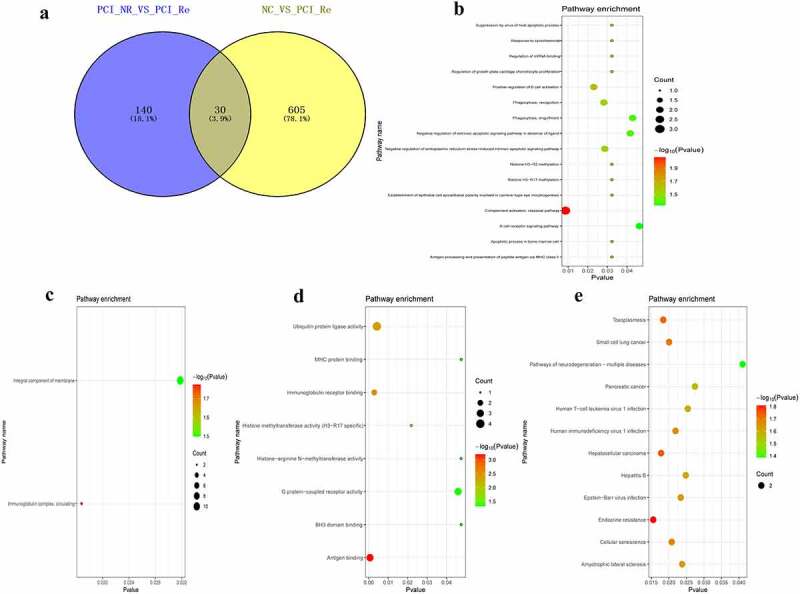
Analysis of DEmRNAs in the intersection of the PCI_NR/PCI_Re and NC/PCI_Re groups.

The intersection of DEmRNAs of the PCI_NR/PCI_Re and NC/PCI_Re groups was established, and a total of 30 genes were obtained (Figure 5a). Moreover, the trend in the expression levels of the aforementioned 30 genes was consistent between the two groups. In order to understand the biological functions of intersection DEmRNAs, GO and KEGG functional analyses were performed (Pval_adj<0.05). The BP, CC and MF enrichment results are displayed in Figure 5b-d. Results of the KEGG enrichment analysis demonstrated that endocrine resistance and hepatocellular carcinoma were significantly enriched signaling pathways (Figure 5e).

a: Venn diagram of DEmRNAs in the PCI_NR/PCI_Re and NC/PCI_Re groups; b: Top 15 significantly enriched biological process (BP); c: Cell composition (CC); d: Molecular function (MF); E: Kyoto Encyclopedia of Genes and Genomes (KEGG) pathways.

### Analysis of DEmRNAs of the intersection of the NC/ACS and ACS/PCI_NR groups

**Figure 6.**
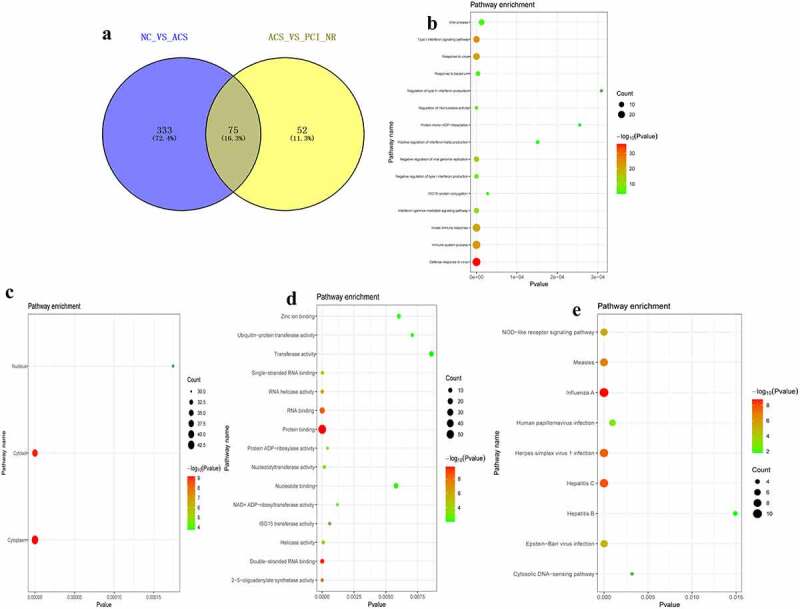
Analysis of DEmRNAs in the intersection of the NC/ACS and ACS/PCI_NR groups.

The intersection of DEmRNAs of the NC/ACS and ACS/PCI_NR groups was established, and a total of 75 genes were obtained (Figure 6a). Moreover, the trend in the expression levels of the aforementioned 75 genes was opposite between the two groups. In order to understand the biological functions of intersection DEmRNAs, GO and KEGG functional enrichment analyses were performed (Pval_adj<0.05). The BP, CC and MF enrichment results are displayed in Figure 6b-d. Results of the KEGG enrichment analysis demonstrated that influenza A and hepatitis C were significantly enriched signaling pathways (Figure 6e).

a: Venn diagram of DEmRNAs in the NC/ACS and ACS/PCI_NR groups; b: Top 15 significantly enriched biological process (BP); c: Cell composition (CC); d: Molecular function (MF); e: Kyoto Encyclopedia of Genes and Genomes (KEGG) pathways.

### Analysis of DElncRNAs in different groups

**Figure 7.**
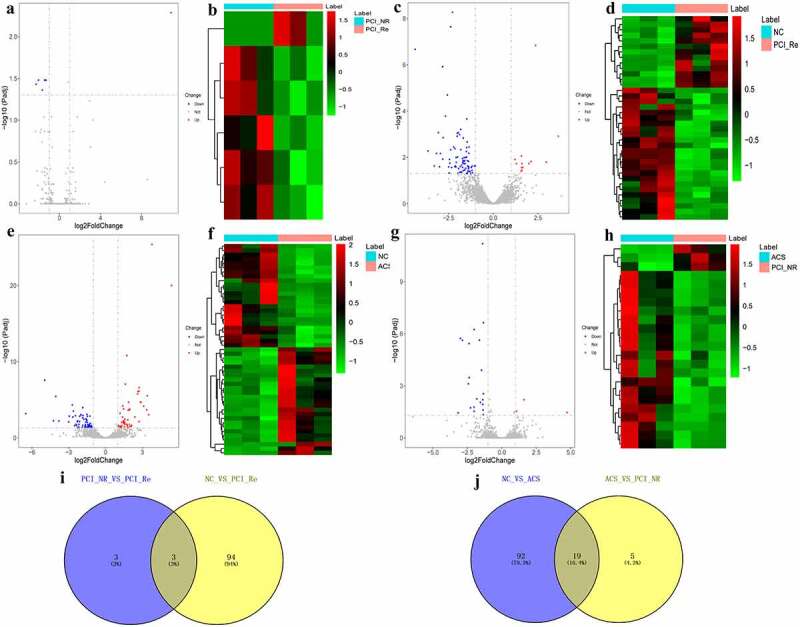
Analysis of DElncRNAs in different groups.

According to the aforementioned screening criteria, there were 6 DElncRNAs (1 up-regulated and 5 down-regulated) in the PCI_NR/PCI_Re group. The volcano map and heat map of DElncRNAs are displayed in Figure 7a and b. Moreover, there were 97 DElncRNAs (13 up-regulated and 84 down-regulated) in the NC/PCI_Re group. The volcano map and heat map of DElncRNAs are displayed in Figure 7c and d. There were 110 DElncRNAs (47 up-regulated and 63 down-regulated) in the NC/ACS group. The volcano map and heat map of DElncRNAs was shown in Figure 7e and f. Results of the present study also demonstrated that there were 24 DElncRNAs (3 up-regulated and 21 down-regulated) in the ACS/PCI_NR group. The volcano map and heat map of DElncRNAs was shown in Figure 7g and h. The intersection of DElncRNAs of the PCI_NR/PCI_Re and NC/PCI_Re groups was established, and a total of 3 genes were obtained (Figure 7i). Moreover, the trend in the expression levels of the aforementioned 3 genes was consistent between the two groups. The intersection of DElncRNAs of the NC/ACS and ACS/PCI_NR groups was established, and a total of 19 genes were obtained (Figure 7j). The trend in the expression levels of the aforementioned 19 genes was opposite between the two groups.

a: Volcano map of DElncRNAs in the PCI_NR/PCI_Re group; b: Heatmap of DElncRNAs in the PCI_NR/PCI_Re group; c: Volcano map of DElncRNAs in the NC/PCI_Re group; d: Heatmap of DElncRNAs in the NC/PCI_Re group; e: Volcano map of DElncRNAs in the NC/ACS group; f: Heatmap of DElncRNAs in the NC/ACS group; g: Volcano map of DElncRNAs in the ACS/PCI_NR group; h: Heatmap of DElncRNAs in the ACS/PCI_NR group; i: Venn diagram of DElncRNAs in the PCI_NR/PCI_RE and NC/PCI_RE groups; J: Venn diagram of DElncRNAs in the NC/ACS and ACS/PCI_NR groups. Heatmap shows result of a two-way hierarchical clustering of DElncRNAs (row) and samples (column). DElncRNAs clustering tree is displayed on the left. Red indicates above the reference channel. Green indicates below the reference channel.

### Co-expression analysis of DEmRNA-DElncRNA in the intersection groups

**Figure 8.**
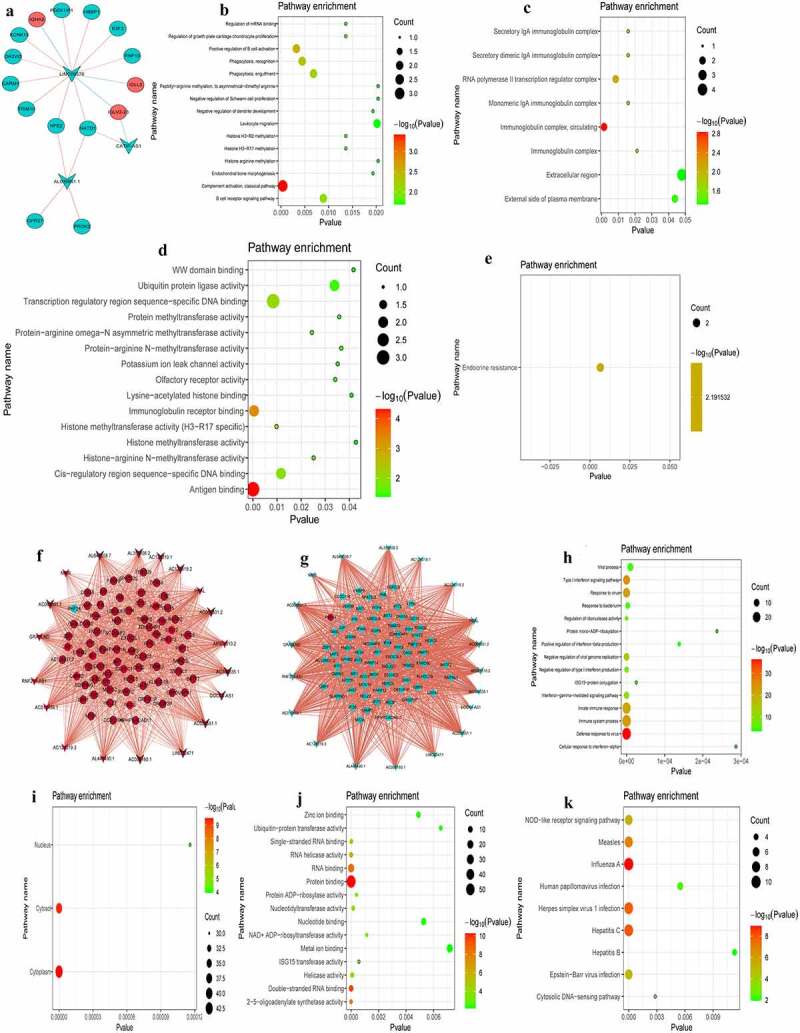
Co-expression analysis of DEmRNA-DElncRNA.

P < 0.01 and |r|≥0.8 were used as the criteria for determining associations between DElncRNA-DEmRNA pairs. Results of the present study demonstrated that there were 19 DElncRNA-DEmRNA pairs (for example, *PROK2-AL035661.1, E2F2-LINC00570*) in the PCI_NR/PCI_Re and NC/PCI_Re intersection group (Figure 8a). In the DEmRNA-DElncRNA co-expression network, 15 DEmRNAs and 3 DElncRNAs were included. Among them, *E2F2, PDZK1IP1, TRIM10, HBBP1, IGLL5, PROK2* and *LINC00570* were the top 10 differentially expressed genes. In order to understand the biological function of DEmRNAs co-represented with DElncRNAs, GO and KEGG functional analyses were performed (Pval_adj<0.05). The BP, CC and MF enrichment results are displayed in Figure 8b-d. Results of the KEGG enrichment analysis demonstrated that endocrine resistance was the only significantly enriched signaling pathway (Figure 8e).

a: DEmRNA-DElncRNA co-expression network in the PCI_NR/PCI_Re and NC/PCI_Re groups; b: Biological process (BP) enrichment of co-expressed mRNA in PCI_NR/PCI_Re and NC/PCI_Re intersection group; c: Cell composition (CC) enrichment of co-expressed mRNA in PCI_NR/PCI_Re and NC/PCI_Re intersection group; d: Molecular function (MF) enrichment of co-expressed mRNA in PCI_NR/PCI_Re and NC/PCI_Re intersection group; e: Kyoto Encyclopedia of Genes and Genomes (KEGG) pathways of co-expressed mRNA in PCI_NR/PCI_Re and NC/PCI_Re intersection group. f: DEmRNAs-DElncRNAs co-expression network in the NC/ACS; g: DEmRNAs-DElncRNAs co-expression network in the ACS/PCI_NR; h: BP enrichment of co-expressed mRNA in the NC/ACS and ACS/PCI_NR intersection group; i: CC enrichment of co-expressed mRNA in the NC/ACS and ACS/PCI_NR intersection group; j: MF enrichment of co-expressed mRNA in the NC/ACS and ACS/PCI_NR intersection group; k: KEGG pathways enrichment of co-expressed mRNA in the NC/ACS and ACS/PCI_NR intersection group. In the DEmRNAs-DElncRNAs co-expression network, the circular, V-shaped, red, green, red lines, and blue lines represent mRNA, lncRNA, up-regulation, down-regulation, positive correlation and negative correlation, respectively.

There were 993 DElncRNA-DEmRNA pairs (for example, *IFI44-NRIR*) in the NC/ACS and ACS/PCI_NR intersection group (figure 8f and g). In the DEmRNA-DElncRNA co-expression network, 74 DEmRNAs and 19 DElncRNAs are included. Among them, *RNF213, LAMP3, IFI44, IFIT1, IFIT3, IFIT5, REC8, XAF1* and *NRIR* were the top 10 differentially expressed genes. In order to understand the biological function of DEmRNAs co-represented with DElncRNAs, GO and KEGG functional analysis were performed (Pval_adj<0.05). The BP, CC and MF enrichment results are displayed in Figure 8h-J. Results of the KEGG enrichment analysis demonstrated that influenza A and hepatitis C were significantly enriched signaling pathways (Figure 8k).

### Diagnostic analysis of DEmRNAs

**Figure 9.**
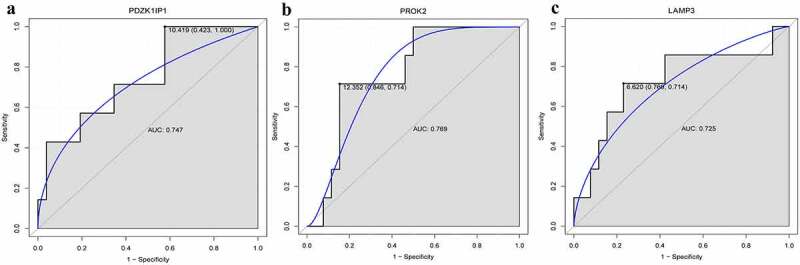
Diagnostic analysis of *PDZK1IP1* (a), *PROK2* (b) and *LAMP3* (c).

We selected the genes in the top 10 of differential expression and also involved in the co-expression of lncRNA-mRNA for diagnostic analysis in the GSE60993 dataset. Results of the ROC curve analysis demonstrated that only the area under curve (AUC) of *PDZK1IP1, PROK2* and *LAMP3* were >0.7, at 0.747, 0.769 and 0.725, respectively (Figure 9). These results indicated that *PDZK1IP1, PROK2* and *LAMP3* may act as the potential diagnostic genetic biomarkers in ACS.

AUC: area under curve

### RT-PCR analysis

**Figure 10.**
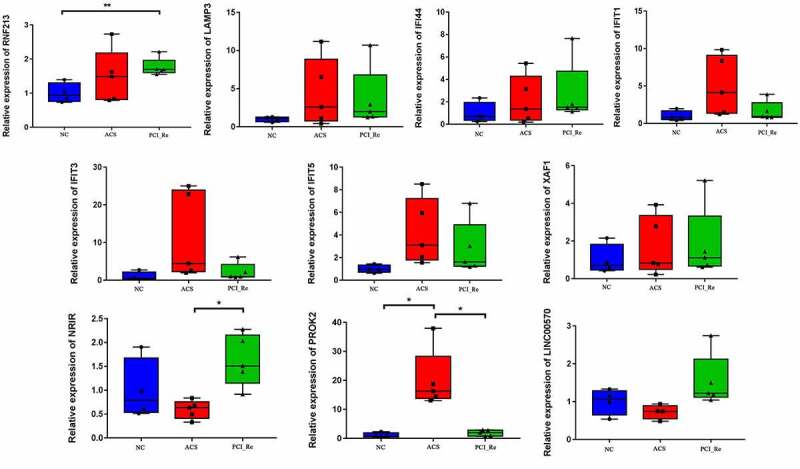
RT-PCR validation of *PROK2, LINC00570, RNF213, LAMP3, IFI44, IFIT1, IFIT3, IFIT5, XAF1* and *NRIR* in blood samples.

The information of 14 individuals is displayed in Table 2. *PROK2, LINC00570, RNF213, LAMP3, IFI44, IFIT1, IFIT3, IFIT5, XAF1* and *NRIR* were selected for RT-PCR validation. All primers used in the present study are shown in Table 3. Results of the RT-PCR analysis demonstrated that compared with normal controls, the expressions levels of *RNF213, LAMP3, IFI44, IFIT1, IFIT3, IFIT5* and *XAF1* were up-regulated in ACS (Figure 10), which was consistent with the results of RNA-seq. However, the expression levels of *NRIR, PROK2* and *LINC00570* were not consistent with the results obtained during RNA-seq analysis. Inconsistency in these results may be due to small sample sizes; thus, further investigations are required.

**Table 2.** The information of 14 individuals for RT-PCR validation

Group	Sample number	Gender	Age	Hypertension history	Diabetes history	Smoking history	Drinking history	Pressure conditions	Atherosclerosis history	Chest pain, chest tightness, arrhythmia, heart failure and other symptoms	Low Density Lipoprotein
Normal controls (NC) group	1	Female	73	No	No	No	No	Small	No	No	2.9 mmol/L
	2	Female	66	No	No	No	No	Small	No	No	3.2 mmol/L
	3	Female	71	No	No	No	No	Small	No	No	2.8 mmol/L
	4	Male	46	No	No	No	No	Small	No	No	2.3 mmol/L
Acute coronary syndrome (ACS) group	1	Male	55	Yes	Yes	Yes	Yes	Big	Yes	No	4.6 mmol/L
	2	Male	58	Yes	No	Yes	Yes	Big	Yes	Yes	3.7 mmol/L
	3	Female	72	No	Yes	No	No	Small	Yes	Yes	4.8 mmol/L
	4	Male	70	Yes	No	Yes	Yes	Big	No	Yes	3.9 mmol/L
	5	Female	69	Yes	No	No	No	Big	Yes	Yes	4.1 mmol/L
Percutaneous coronary intervention_restenosis (PCI_Re) group	1	Male	75	Yes	Yes	No	Yes	Small	Yes	Yes	4.2 mmol/L
	2	Male	77	Yes	No	Yes	Yes	Big	Yes	No	3.2 mmol/L
	3	Female	70	Yes	Yes	No	No	Small	Yes	Yes	4.3 mmol/L
	4	Male	72	Yes	Yes	Yes	Yes	Small	Yes	Yes	4.8 mmol/L
	5	Male	59	No	Yes	Yes	Yes	Big	No	Yes	4.1 mmol/L

Group		High density lipoprotein	Total cholesterol	Triglyceride	Blood platelet count	Cardiactroponin (cTn)	Creatine kinase isoenzyme(CK-MB)	Electrocardiograph	Percutaneous coronary intervention (PCI) treatment	Time for stenosis after PCI	Coronary angiography results (percentage of restenosis in the original lesion)
Normal controls (NC) group		1.4 mmol/L	3.8 mmol/L	2.2 mmol/L	228	0.01 ug/L	8 U/L	Normal			
		1.5 mmol/L	4.5 mmol/L	1.5 mmol/L	247	0.02 ug/L	14 U/L	Normal			
		1.8 mmol/L	5.6 mmol/L	1.9 mmol/L	254	0.01 ug/L	11 U/L	Normal			
		1.2 mmol/L	4.1 mmol/L	2.1 mmol/L	221	0.03 ug/L	12 U/L	Normal			
Acute coronary syndrome (ACS) group		1.2 mmol/L	6.8 mmol/L	2.6 mmol/L	225	0.4 ug/L	22 U/L	ST-T changed			
		1.3 mmol/L	6.5 mmol/L	3.7 mmol/L	126	2.5 ug/L	46 U/L	ST-T changed			
		1.1 mmol/L	7.1 mmol/L	3.1 mmol/L	256	0.3 ug/L	12 U/L	ST-T changed			
		1.5 mmol/L	6.6 mmol/L	2.7 mmol/L	187	0.4 ug/L	25 U/L	ST-T changed			
		1.6 mmol/L	7.2 mmol/L	2.1 mmol/L	207	0.2 ug/L	20 U/L	ST-T changed			
Percutaneous coronary intervention_restenosis (PCI_Re) group		1.1 mmol/L	6.2 mmol/L	2.2 mmol/L	210	2.8 ug/L	66 U/L	ST-T changed	2 anterior descending brace	3 months	85%
		1.1 mmol/L	6.3 mmol/L	2.4 mmol/L	214	0.09 ug/L	17 U/L	ST-T changed	1 diagonal brace, 2 right crown braces	6 months	96%
		1.1 mmol/L	6.5 mmol/L	4.3 mmol/L	258	0.05 ug/L	9 U/L	ST-T changed	1 anterior descending branch open stent, 1 right crown middle stent	10 months	80%
		1.4mmol/L	6.6mmol/L	2.1mmol/L	187	0.06ug/L	10U/L	ST-T changed	2 anterior descending stents, 1 right crown stent	12 months	90%
		1.5mmol/L	7.2mmol/L	2.9mmol/L	245	0.1ug/L	18U/L	ST-T changed	2 circumflex supports	1 month	95%

**Table 3.** Primer sequence in the RT-qPCR

Primer name	Primer sequence (5ʹ to 3ʹ)
GAPDH-F (internal reference)	5-CTGGGCTACACTGAGCACC-3
GAPDH-R (internal reference)	5-AAGTGGTCGTTGAGGGCAATG-3
ACTB-F (internal reference)	5-GATCAAGATCATTGCTCCTCCT-3
ACTB-R (internal reference)	5-TACTCCTGCTTGCTGATCCA-3
RNF213-F	5-GCTGCTGTGAAAAACGAGAAG-3
RNF213-R	5-TCCCATTTTGACTCCCCAAATTC-3
LAMP3-F	5-AGCAAGCACCTCACCAAACTT-3
LAMP3-R	5-TGTAGTCGCTGGGGTAGTTGT-3
IFI44-F	5-GGTGGGCACTAATACAACTGG-3
IFI44-R	5-CACACAGAATAAACGGCAGGTA-3
IFIT1-F	5-TCAGCACTTCGATGGGACG-3
IFIT1-R	5-ACACTGCAGCCTCGAACTC-3
IFIT3-F	5-AAAAGCCCAACAACCCAGAAT-3
IFIT3-R	5-CGTATTGGTTATCAGGACTCAGC-3
IFIT5-F	5-CCGGAAAGCTCTTCGTCTGG-3
IFIT5-R	5-TGCGAAGGGGTGATCTGTCT-3
XAF1-F	5-GCTCCACGAGTCCTACTGTG-3
XAF1-R	5-GTTCACTGCGACAGACATCTC-3
NRIR-F	5-TCTGTCGCCAGGCTGGAGTG-3
NRIR-R	5-GGCTGAGGCAGGATAATCGCTTG-3
PROK2-F	5-CTGCCATCCACTGACTCGT-3
PROK2-R	5-GTCCGTAAACAGGCCAAGC-3
LINC00570-F	5-GGGGATCAACGAACAGGCT-3
LINC00570-R	5-ACTCAGTCTCCAGCACTCCT-3

* represent P < 0.05, ** represent P < 0.01, P < 0.05 was considered significant. NC, normal control; ACS, acute coronary syndrome; PCI_Re, percutaneous coronary intervention_ restenosis.

## Discussion

The mechanisms underlying ACS progression are mediated by multiple molecules [37–40], and numerous previous studies have based their research on the use of public databases or certain molecules for the study of ACS. Notably, ACS and in-stent restenosis have rarely been studied at the level of the transcriptome. The blood transcriptome reflects the state of the disease [41]. However, there are few studies on the identification of novel biomarkers from the peripheral blood transcriptome of patients with ACS. Bioinformatics analysis is a common method to identify potential key molecule biomarkers and signaling pathways in diseases [42–45]. Thus, the present study aimed to identify key molecular markers of ACS and in-stent restenosis from peripheral blood using transcriptome sequencing and bioinformatics analysis. Studying the expression and interaction of ACS and in-stent restenosis-associated genes is of great significance for understanding the molecular mechanisms underlying ACS, and provides a novel theoretical basis for the diagnosis and treatment of ACS.

In the present study, there were 15 DEmRNAs and 3 DElncRNAs in the PCI_NR/PCI_Re and NC/PCI_Re intersection group. We selected genes in the top 10 of differential expression and also involved in the co-expression of lncRNA-mRNA for related discussions. Results of a previous study demonstrated that compared with wild-type mice, *E2F transcription factor 2* (*E2F2*) deficient mice exhibited abnormal blood vessels and elevated blood pressure [46]. *E2F2* induces the proliferation of cardiomyocytes, and reduces the expression of pro-apoptotic genes [47,48]. Results of the present study highlighted that the expression of *E2F2* was significantly altered, and was co-expressed with *LINC00570*. Therefore, we hypothesized that *E2F2* and *LINC00570* play an important regulatory role in the progression of ACS through co-expression regulation of angiogenesis and proliferation of cardiac myocytes. Results of a previous study found demonstrated *Prokineticin 2* (*PROK2*) (also known as *PK2*) alleviated H9C2 myocardial cell injury induced by hypoxia/reoxygenation (H/R) through the activation of the Akt/mTOR pathway [49]. *PROK2* also inhibits high glucose/high palmitic acid-induced apoptosis of cardiomyocytes by inhibiting oxidative stress and autophagosome accumulation [50]. In addition, *PROK2* plays a role in angiogenesis and inflammation [51,52]. Through lncRNA-mRNA co-expression network, results of the present study revealed that *PROK2* was co-expressed with *AL035661.1*. Thus, we hypothesized that the roles of *PROK2* in angiogenesis, and proliferation and apoptosis of cardiac myocytes may be regulated by *AL035661.1*, thus affecting the progression and recurrence of ACS. In addition, the AUC of *PROK2* was >0.7, which indicated that *PROK2* may be a potential diagnostic biomarker of ACS.

Few studies have revealed the role of *PDZK1 interacting protein 1* (*PDZK1IP1*) in cardiovascular diseases; however, PDZK1IP1 acts as a potential biomarker and is abnormally expressed in tumors [53,54]. *Tripartite motif containing 10* (*TRIM10*) is also associated with immune response [55], and regulates cardiac hypertrophy through the PTEN/AKT pathway [56]. *Hemoglobin subunit beta pseudogene 1* (*HBBP1*) plays an important role in erythropoiesis and β-thalassemia [57]. *Immunoglobulin lambda like polypeptide 5* (*IGLL5*) is up-regulated in inflammatory diseases [58,59]. In addition, results of the KEGG enrichment analysis demonstrated that *IGLL5* was associated with primary immunodeficiency and chemokine signaling pathways [58]. To the best of our knowledge, the roles of *PDZK1IP1, TRIM10, HBBP1* and *IGLL5* have not previously been reported in ACS, and the present study may be the first to report the potential molecular regulatory role of the aforementioned genes in ACS. Results of the lncRNA-mRNA co-expression network used in the present study demonstrated that *PDZK1IP1, TRIM10, HBBP1* and *IGLL5* were co-expressed with *LINC00570*. However, to the best of our knowledge, the role of *LINC00570* is yet to be reported in cardiovascular disease. Further investigation into the roles of *PDZK1IP1, TRIM10, HBBP1, IGLL5* and *LINC00570* will provide novel insights into the pathogenesis of ACS, and may contribute to the development of potential diagnostic and therapeutic targets for ACS. Notably, results of the present study revealed that the AUC of *PDZK1IP1* was >0.7, which indicated that *PDZK1IP1* may be a potential diagnostic biomarker of ACS.

Moreover, results of the present study revealed a large number of DEmRNAs and DElncRNAs in the NC_VS_ACS and ACS_VS_PCI_NR intersection group. We selected genes in the top 10 of differential expression and also involved in the co-expression of lncRNA-mRNA for related discussions. *Interferon induced protein with tetratricopeptide repeats* (*IFIT*) *1, IFIT3* and *IFIT5* belong to the IFIT family of genes [60]. Up-regulation of IFITs expression is critical for cardiomyocyte clearance of coxsackievirus B3 (CVB3) and the prevention of myocarditis [61]. Moreover, increased expression of *IFIT1* will promote inflammation, and *IFIT1* can be used as a potential target for alleviating atherosclerosis-related diseases [62]. *IFIT3* may be a potential therapeutic target for ischemic cardiomyopathy [63], and *IFIT1* and *IFIT5* are key genes that are used to predict treatment response in patients with microvascular disease in the early stage [64]. Previous studies have demonstrated that *IFIT1, IFIT3* and *IFIT5* play an important regulatory role in cardiovascular disease. In the present study, the expression levels of *IFIT 1, IFIT3* and *IFIT5* were significantly altered, and were co-expressed with multiple DElncRNAs. Thus, we hypothesized that *IFIT1, IFIT3* and *IFIT5* may co-regulate the occurrence of ACS together with related DElncRNAs. Identification of *IFIT1, IFIT3* and *IFIT5* in the present study contributes to the discovery of potential molecular biomarkers for ACS, and also provides direction for further understanding the pathogenesis of ACS. In addition, results of the KEGG functional enrichment analysis in the present demonstrated that *IFIT1* was associated with hepatitis C. Patients with hepatitis C virus (HCV) infection have a greater risk of developing ACS than those without HCV infection [65]. Compared with patients who are not infected with HCV, patients with ACS and HCV infection have increased platelet reactivity, coronary heart disease is more serious, and the prognosis is worse [66]. Notably, successful HCV treatment significantly reduced the incidence of ACS in patients with type 2 diabetes [67]. Therefore, *IFIT1* may regulate the progression of ACS by regulating hepatitis C.

*Ring finger protein 213* (*RNF213*) is a susceptibility gene of moyamoya disease and plays an important role in vascular development [68,69]. Overexpression of the vascular endothelial cell-specific *RNF213* mutant aggravated the hypoxia-induced PH phenotype (high right ventricular pressure, right ventricular hypertrophy, and pulmonary vascular muscularization) [70]. Notably, results of a previous study demonstrated that the *RNF213* p.R4810K variant was significantly associated with coronary artery disease [71]. Moreover, previous studies have also demonstrated that *lysosomal associated membrane protein 3* (*LAMP3*) is expressed in human heart and cardiomyocytes, and that its expression is elevated in dilated cardiomyopathy hearts with severe heart remodeling [72]. *Interferon induced protein 44* (*IFI44*) may be involved in vascular lesions and pathogenesis of systemic sclerosis (SSc) [73], and is also associated with immune and inflammatory diseases [74,75]. *REC8 meiotic recombination protein* (*REC8*) inhibits tumor angiogenesis by inhibiting NF-κB mediated expression of vascular endothelial growth factor [76]. *XIAP associated factor 1* (*XAF1*) is a pro-apoptotic factor, and knockdown of *XAF1* inhibits the apoptosis of vascular smooth muscle cells induced by interferon (IFN)-gamma [77]. Overexpression of *XAF1* induces cell apoptosis and has potential anti-angiogenesis effects [78]. ACS describes a group of severe cardiovascular diseases, and includes acute myocardial infarction and unstable angina [1,2]. Thus, we hypothesized that *RNF213, LAMP3, IFI44, REC8* and *XAF1* may play key roles in the progression of ACS by regulating vascular lesions. In the present study, the expression levels of *RNF213, LAMP3, IFI44, REC8* and *XAF1* were significantly altered, and were co-expressed with multiple DElncRNAs. For example, *IFI44* and *Negative regulator of interferon response* (*NRIR*) was a co-expression pair. *NRIR*, also known as *lncCMPK2* or *lncRNA-CMPK2*, was significantly up-regulated in SSc monocytes and NRIR expression was associated with the IFN score of patients with SSc [79]. Moreover, *NRIR* is abnormally expressed in the peripheral blood mononuclear cells of patients with Sjogren’s syndrome, and it is closely associated with the mRNA functions in immune response and cell metastasis [80]. Therefore, we speculated that *RNF213, LAMP3, IFI44, REC8* and *XAF1* may co-regulate the occurrence of ACS together with related DElncRNAs. Identification of *RNF213, LAMP3, IFI44, REC8, XAF1* and *NRIR* in the present study contributes to the discovery of potential molecular biomarkers for ACS, and also provides direction for further understanding the pathogenesis of ACS. In addition, the AUC of *LAMP3* was >0.7, which indicates that *LAMP3* may be a potential diagnostic biomarker of ACS.

Dataset GSE60993 was blood transcriptome sequencing data of patients with ACS [41]. Previous studies have identified key genes and signaling pathways associated with ACS progression in the GSE60993 dataset through bioinformatics analysis [81–84]. Numerous previous studies have relied on the use of public databases for analysis. In the present study, patients with ACS, PCI_NR patients, PCI_Re patients and healthy controls were enrolled for transcriptome sequencing analysis, and genes associated with ACS and recurrence following treatment were screened. For further verification, we selected the genes in the top 10 of differential expression and also involved in the co-expression of lncRNA-mRNA for diagnostic analysis in the GSE60993 dataset. Results of the present study revealed that *PDZK1IP1, PROK2* and *LAMP3* may act as potential biomarkers of ACS and recurrence following treatment, and the expression of these genes may act as biomarkers for early medical intervention of the disease.

However, the present study has limitations. Firstly, the sample sizes used for sequencing and RT-PCR analyses were small. Thus, the sample size of each group must be increased to further verify the results obtained. Moreover, the molecular mechanisms underlying the regulation of the identified genes in ACS and PCI_Re remain to be fully elucidated, and further investigations are required.

## Conclusion

In the present study, numerous DEmRNAs and DElncRNAs were identified through transcriptome sequencing. Results of the present study indicated that the identified mRNAs and lncRNAs may be used as potential clinical biomarker for ACS. Although the present study has limitations, ACS transcriptome sequencing analysis and the construction of mRNA-lncRNA co-expression network provide potential targets for the diagnosis and treatment of ACS and PCI_Re, and also provide a novel theoretical basis for future studies. Collectively, results of the present study demonstrated that *PDZK1IP1, PROK2* and *LAMP3* may act as potential biomarkers of ACS.

## Data Availability

The datasets generated during and/or analysed during the current study are available from the corresponding author on reasonable request. The transcriptome data have been uploaded to Gene Expression Omnibus (accession no. GSE179645).

## Highlights

- Transcriptome sequencing analysis provides potential biomarkers for the diagnosis and treatment of ACS.
- *PDZK1IP1, PROK2* and *LAMP3* may act as the potential diagnostic genetic biomarkers in ACS.
- Identification of potential key genes provides direction for further research.
